# Relationship of Creatinine and Nutrition with Arsenic Metabolism: Smith et al. Respond

**DOI:** 10.1289/ehp.1104807R

**Published:** 2012-04-01

**Authors:** Allan H. Smith, Jane Liaw, Craig Steinmaus, Arin Basu, Soma Mitra, David Kalman

**Affiliations:** Arsenic Health Effects Research Group, School of Public Health, University of California, Berkeley, Berkeley, California, USA, E-mail: ahsmith@berkeley.edu; Health Sciences Centre, University of Canterbury, Christchurch, New Zealand; School of Human Sciences, London Metropolitan University, London, United Kingdom; Department of Environmental Health, University of Washington, Seattle, Washington, USA

We thank Hall and Gamble for their interest in our findings. In our article (Basu et al. 2011), we cited their earlier results concerning urinary dimethylarsinic acid (DMA) and creatinine concentrations, noting that in 2005 they reported a strong correlation between urinary creatinine and the percentage of DMA (DMA%) (Gamble et al. 2005). We also noted rather similar findings from another group working in Bangladesh (Nermell et al. 2008), so together with our findings in India, there are three separate studies that have reported this association (Basu et al. 2011; Gamble et al 2005; Nermell et al 2008). Our results highlight the strength of the relationship between urinary creatinine and urinary DMA%, which was stronger than the relationship between DMA% and any of the 19 dietary factors and 16 blood micronutrients that we investigated.

Gamble and Hall state that they had previously reported (Gamble and Liu 2005) that one implication of the observed association between urinary creatinine and arsenic methylation was that urinary As should not be expressed as per gram creatinine to correct for urine concentrations. This is not quite what they said, although they did point out risks in expressing urinary arsenic per gram of creatinine. However, in that letter (Gamble and Liu 2005) and again in this one, they state that urinary creatinine should be included as a covariate in regression models. We do not agree with this recommendation. Steinmaus et al. (2009) reported a spurious relationship between low-level arsenic concentrations and diabetes in the United States after including urinary creatinine in a regression model. They noted that urinary creatinine concentrations may change as a consequence of diabetes, which is known to affect renal function, and that it is not appropriate to adjust for a factor that is a consequence of the disease being studied.

We also do not agree with Gamble and Hall’s criticism of our blood sample handling, which they appear to have raised because our folate findings were different from theirs (e.g., Gamble et al. 2005). For our study (Basu et al. 2011), blood samples were collected in remote villages, and on occasion they had to be stored on ice for up to 24 hr. Gamble and Hall state that “this 24-hr delay can be problematic for some nutrients, especially folate, which is extremely sensitive to oxidative degradation.” In support of this statement they cite Drammeh et al. (2008), who found reductions in serum folate concentrations (about 30%) from whole blood stored for 1 day at 32°C. However, storing blood samples on ice at about 0°C is not the same as storing at 32°C, so this result is not relevant. In addition, Drammeh et al. cited a study that reported stable plasma folate levels during storage of up to 7 days at 4°C (Kubasik et al. 1979). We therefore do not think our findings should be criticized based on sample storage.

However, this does not mean we suggest that folate is not related to DMA%. Findings may vary from study to study, and we accept that Gamble et al. have reported several studies suggesting that folate is associated with small increases in arsenic methylation, including a randomized trial with folate supplements (Gamble et al. 2006). In a study of pregnant women in Bangladesh, a small association between folate and arsenic methylation was reported (Gardner et al. 2011; Li et al. 2008); the authors concluded that nutritional status has a marginal influence on the metabolism of arsenic in pregnant women. Although the overall evidence suggests that folate may increase arsenic methylation, the magnitude of the effect is small and in our opinion of little consequence.

In their letter Gamble et al. report they are now conducting a randomized trial with creatine. Well-run randomized trials of nutritional supplements with sufficient statistical power typically require a large amount of public health funds and resources. We suggest that randomized trials should be conducted only on people whose exposure to arsenic has ceased. However, it is clear that exposures did not cease in the randomized trial of folate they conducted, but that exposures remained markedly elevated based on urine arsenic concentrations (Gamble et al. 2006). The purpose of a randomized trial of folate or creatine is to determine whether these agents might affect arsenic methylation to help reduce arsenic toxicity and increase excretion. However, the relevance of this goal is questionable because we already know the best and most appropriate way to reduce arsenic toxicity—by reducing exposure. The first priority should be to stop exposure to arsenic. If exposure to arsenic ceased, there would be very little arsenic to methylate and enhancement of methylation with supplements would not be necessary.
